# A non‐canonical Receptor tyrosine kinase DDR2 resides inside the nuclear speckles and regulates alternative splicing in Alzheimer’s

**DOI:** 10.1002/alz.088623

**Published:** 2025-01-03

**Authors:** Russa Das, Debashis Mukhopadhyay

**Affiliations:** ^1^ Homi Bhabha National Institute, Mumbai, Maharashtra India; ^2^ Saha Institute of Nuclear Physics, Kolkata, West Bengal India

## Abstract

**Background:**

Receptor Tyrosine kinase‐mediated signaling is indispensable for the cell’s normal functioning, the perturbation of which leads to disease conditions. The altered expression and activity of several Receptor Tyrosine kinases (RTKs) are known to regulate the pathophysiology of Alzheimer’s disease (AD). However, the mechanistic details remain illusive. Discoidin Domain Receptor 2 (DDR2) is a unique set of RTKs that gets significantly upregulated in an Alzheimer’s disease (AD) cell model and AD brain lysate.

**Method:**

The AD cell model was prepared using SH‐SY5Y cells and treated with AICD and Aß for 48 hours and was checked for the activity and expression level of DDR2. The expression level of the collagens was also checked. Molecular docking studies were performed to predict the interaction between DDR2 and Amyloid beta. ICC followed by nuclear fractionation studies was performed to confirm the compartmental distribution of DDR2 in the AD model. Immunoprecipitation followed by mass‐spectrometry was done to confirm the interactors of DDR2 in AD.

**Result:**

The mRNA and protein levels and activity of DDR2 were significantly upregulated in the AD cell model. There were no significant changes in the mRNA expression level of collagens. Molecular docking followed by ICC studies confirms the interaction between DDR2 and amyloid beta. Again, for the first time, we showed that DDR2 translocates to the nucleus in an untreated condition in SH‐SY5Y cells, and also the DDR2 level increases in AD nucleus compared to control using ICC and nuclear fractionation. Moreover, we observed that DDR2 forms a punctate structure inside the nucleus that strongly colocalizes with SRSF2 a nuclear speckle protein. Immunoprecipitation of DDR2 followed by Mass spectrometry (MS) in the AD cell model revealed some important proteins like HNRNPH1, HNRNPU, PCBP2, and SRSF7 as binding partners of DDR2, implicating a role of DDR2 in mRNA splicing.

**Conclusion:**

DDR2 which is a receptor tyrosine kinase, in the absence of its endogenous ligand collagen gets activated in AD by interacting with amyloid beta and translocates to the nucleus. DDR2 resides inside the nuclear speckles, and interacts with several splicing factors, thereby regulating alternative splicing in AD.

